# The genomic study of an environmental isolate of *Scedosporium apiospermum* shows its metabolic potential to degrade hydrocarbons

**DOI:** 10.1186/s40793-017-0287-6

**Published:** 2017-12-04

**Authors:** Laura T. Morales, Laura N. González-García, María C. Orozco, Silvia Restrepo, Martha J. Vives

**Affiliations:** 1 Centro de Investigaciones Microbiológicas, Department of Biological Sciences, Universidad de los Andes, Bogotá, Colombia; 2 Laboratorio de Micología y Fitopatología Uniandes, Universidad de los Andes, Bogotá, Colombia; 3 Department of Biological Sciences, Universidad de los Andes, Bogotá, Colombia

**Keywords:** *Scedosporium apiospermum*, Genome, Hydrocarbon degradation

## Abstract

Crude oil contamination of soils and waters is a worldwide problem, which has been actively addressed in recent years. Sequencing genomes of microorganisms involved in the degradation of hydrocarbons have allowed the identification of several promoters, genes, and degradation pathways of these contaminants. This knowledge allows a better understanding of the functional dynamics of microbial degradation. Here, we report a first draft of the 44.2 Mbp genome assembly of an environmental strain of the fungus *Scedosporium apiospermum.* The assembly consisted of 178 high-quality DNA scaffolds with 1.93% of sequence repeats identified. A total of 11,195 protein-coding genes were predicted including a diverse group of gene families involved in hydrocarbon degradation pathways like dioxygenases and cytochrome P450. The metabolic pathways identified in the genome can potentially degrade hydrocarbons like chloroalkane/alkene, chorocyclohexane, and chlorobenzene, benzoate, aminobenzoate, fluorobenzoate, toluene, caprolactam, geraniol, naphthalene, styrene, atrazine, dioxin, xylene, ethylbenzene, and polycyclic aromatic hydrocarbons. The comparison analysis between this strain and the previous sequenced clinical strain showed important differences in terms of annotated genes involved in the hydrocarbon degradation process.

**Electronic supplementary material:**

The online version of this article (10.1186/s40793-017-0287-6) contains supplementary material, which is available to authorized users.

## Introduction

Accidental spills of oil have risen as a global important problem due to the serious environmental damages caused by soil and water contamination [1]. Whereas oil is a complex mixture of aromatic and aliphatic hydrocarbons of different molecular weights, its removal from the environment is difficult and its permanence is prolonged [2]. These compounds have gained considerable attention because of their harmful features like resistance to degradation, bioaccumulation, and carcinogenic activity. Their persistence in the environment increases with their molecular weight and there is a need to develop technologies or processes able to degrade or to transform these compounds into less toxic molecules [3].

The ability of several organisms, primarily microorganisms (bacteria, fungi and microalgae), to degrade these toxic substances has been extensively studied in recent decades [1, 4–9]. The main goal is to improve the decontamination of the environment via bioremediation, which encompasses technologies that allow the transformation of compounds to less harmful or not harmful forms, with less use of chemicals, energy, and time [10, 11]. Microbial bioremediation is very effective due to the catabolic activity of microorganisms; among these, many species of bacteria, fungi, and microalgae have demonstrated the ability of hydrocarbon degradation. This process involves the breakdown of organic molecules through biotransformation in less complex metabolites, or mineralization to water, carbon dioxide, or methane [3].

Several strategies have been employed to study these microorganisms and to understand the processes carried out by them. Within these, genomics have allowed the recognition of promoters, genes, and degradation pathways that influence the construction of more efficient degradative strains relevant in bioremediation processes [12, 13]. Genome sequencing of hydrocarbon-degrader organisms has allowed the identification of several genes involved in metabolism and catabolism of aliphatic, aromatic alcohols, and other similar compounds, as well as some metals resistance genes [14]. However, the number of sequenced genomes of fungal species is lower than in bacteria. To date, there are 103,076 prokaryotic genomes sequenced whereas there are only 4503 genomes from eukaryotes in GenBank database (July 2017).

*Scedosporium apiospermum* (teleomorph: *Pseudallescheria* *apiosperma* [15]) is a fungus belonging to the phylum Ascomycota, which has been isolated from various environments, usually in those influenced by human activity [16]. This fungus was reported as a hydrocarbon-degrading microorganism since 1998 due to its ability to degrade polluting compounds, such as phenol and p-cresol [17]. One year later, its ability to degrade phenylbenzoate and its derivatives was elucidated [18]. In recent years, studies regarding degradation of complex compounds, such as toluene [19], polycyclic aromatic hydrocarbons (PAHs) [20], long-chain aliphatic hydrocarbons, and mixtures of these contaminants (unpublished results from our group) [21] have risen. Additionally, the fungus’ ability to regenerate granular activated carbon once it has been saturated with phenol was shown in our laboratory (unpublished results).

Therefore, *Scedosporium apiospermum* presents a wide range of opportunities in bioremediation and its genome sequencing can allow the identification of promoters, genes, and degradation pathways of hydrocarbons. Indeed, the genomic analysis of this fungus can improve the understanding of functional dynamics of contaminants microbial degradation and enhance conditions for effective decontamination processes in different environments [2]. On the other hand, this fungus has been recognized as a potent etiologic agent of severe infections in immunocompromised and occasionally in immunocompetent patients [22]. For this reason, in 2014, the genome of an isolate from a cystic fibrosis patient (clinical strain) was sequenced with the aim of gaining knowledge of its pathogenic mechanisms [23].

Thus, our objective was the complete characterization of the genome of the *S. apiospermum* environmental strain HDO1. In order to analyze the genes and pathways involved in the degradation process and to assess the unique components of its genome compared to the clinical strain and other sister species, we sequenced, assembled, annotated, and fully characterized the environmental strain’s genome.

## Organism information

### Classification and features

**Fig. 1:**
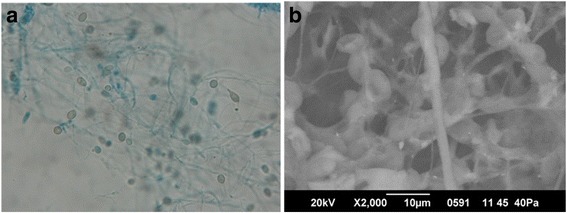
Micrograph of *Scedosporium apiospermum*. **a** Optical microscopy of hyphae and conidia from a PDA culture, at 100× total magnification. Lactophenol cotton blue wet mount preparation. **b** Scanning electron microscopy of hyphae and conidia from a liquid culture grown in minimal salt medium plus crude oil as the sole carbon and energy source

*Scedosporium apiospermum* environmental strain HDO1 was isolated as a contaminant from assays on bacterial strains able to grow in crude oil (API gravity 33) as the unique carbon source. It was selected for sequencing due to its capability to grow in cultures containing aliphatic hydrocarbons of crude oil, naphthalene, phenanthrene, phenol, and mixtures of these compounds in the laboratory. The fungal isolate was grown on potato dextrose agar (OXOID LTD, Hampshire, UK) plates for a period of 7 days at 30 °C. The optimal growth temperature was 30–37 °C. Identification was based on the following morphological characteristics: obverse and reverse colony color (according to the color chart Küppers, H. [24]), colony texture, size, and presence of diffusible pigments, hyphae characteristics, and conidia arrangement. The morphological characteristics were: colonies with a diameter of 7 cm on PDA at 25 °C after 7 days, cottony textured, greyish-white (N00, C00-A00) with yellowish-white reverse. No diffusible pigment was observed. The mycelium was hyaline, septate, and thin. Unbranched conidiphores with long neck-bottle shaped phialides were observed. Conidia were hyaline, approximately 5 μm in diameter, occurring in basipetal chains leaving long hyaline annelids (Fig. 1). For the molecular characterization, the fungus was grown in Sabouraud broth at 25 °C, 150 rpm for 7 days, and the biomass obtained was lyophilized for at least 12 h. Fungal genomic DNA was extracted from 100 mg of lyophilized and pulverized mycelia conducting the CTAB and Phenol/Chloroform/Isoamylic alcohol method [25]. The universal primers used for amplification of the ITS region, were ITS4 (5′-TCCTCCGCTTATTGATATGC-3′) and ITS5 (5′-GGAAGTAAAAGTCGTAACAAGG-3′) [26]. Sanger sequencing was performed by Macrogen (South Korea). Nucleotide sequences obtained were compared with the non-redundant database of the National Center for Biotechnology Information (NCBI) using the tBlastx program (parameters by default), and the ITS region sequences were assigned to the fungus *Scedosporium apiospermum* with an E-value equal to 0.0, 100% query coverage and 100% identity. The obtained sequence is deposited at the NCBI Genbank nr database with the accession number JQ003882.1.

**Fig. 2:**
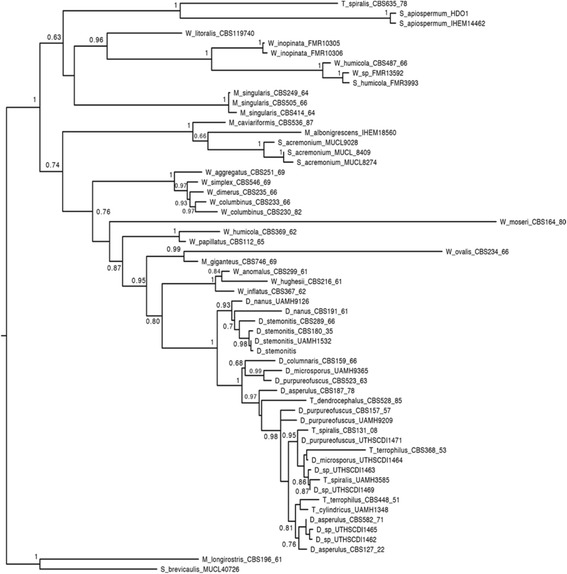
Phylogenetic Analysis of *S. apiospermum* HDO1. Estimated relationships of *S. apiospermum* HDO1 with *S. apiospermum* IHEM 14462 and other species from the *Microascaceae* family. The tree shows the concatenated analysis of the Internal Transcribed Spacer, the Large Subunit and the Elongation factor gene regions. Sequences from reference strains were used (Additional file 1:Table S3). Support values represent Bootstrap support values (Maximum Likelihood)

A phylogenetic analysis was performed using the long subunit rRNA gene, the internal transcribed spacer and the elongation factor 1-α sequences obtained from GenBank. Species from the Microascaceae family were included [27] [28] and are described in the Additional file 1: Table S3. Individual gene regions (*LSU*, *ITS* and *TEF*) were aligned using MAFFT v. 7.187 [29]. Maximum Likelihood analyses were performed using RAxML v.7.6.3 [30] as implemented on the CIPRES portal [31]. The sequence alignment was partitioned into three subsets, each one under a specified model of nucleotide substitution, chosen with PartitionFinder [32]. Estimation of different shapes, GTR rates, and base frequencies for each partition were allowed. The majority rule criterion implemented in RAxML [33] (−autoMRE) was used to assess clade support by bootstrap. The resulting trees were plotted using FigTree v. 1.4.2 [34]. *Microascus longirostris* and *Scopulariopsis brevicaulis* were used as outgroups. Environmental strain HDO1 used in this study clustered with the clinical strain 10.1601/strainfinder?urlappend=%3Fid%3DIHEM+14462 with good support, and they are the sister group of *Trichurus spiralis* 10.1601/strainfinder?urlappend=%3Fid%3DCBS+635.78 (Fig. 2). The whole group is contained within the *wardamycopsis* lineage described by Sandoval-Denis, M. et al. [28]. Summary of the classification and general features of *S. apiospermum* is given in Table 1.

**Table 1:** Classification and general features of *Scedosporium apiospermum* strain HDO1

MIGS ID	Property	Term	Evidence code^a^
	Classification	Domain *Fungi*	TAS [71]
		Phylum *Ascomycota.*	TAS [72]
		Class *Sordariomycetes*	TAS [72]
		Order *Microascales*	TAS [72]
		Family – Microascaceae	TAS [72]
		Genus *Scedosporium*	TAS [73]
		Species *Scedosporium apiospermum*	TAS [73]
		strain: HDO1	
	Gram stain	n/a	
	Cell shape	Mycelium with septae	IDA
	Motility	non-motile	IDA
	Sporulation	Conidia	TAS [72]
	Temperature range	25–42 °C	TAS [17]
	Optimum temperature	30–37 °C	TAS [17]
	pH range; Optimum	5.5–8.5: 7.3	TAS [17]
	Carbon source	Glucose, sucrose, urea, ethanol, ribitol, xylitol, L-arabinitol, phenol, p-cresol, hydroquinone, 1,2,4-benzenetriol, catechol, 4-hydroxybenzylalcohol, 4-hydroxybenzaldehyde,, 4-hydroxybenzoate, protocatechuate, 3-oxoadipate, phenylbenzoate, naphthalene, pyrene, phenantrene, crude oil.	TAS [17–21]
MIGS-6	Habitat	Soil and polluted water	TAS [74, 75]
MIGS-6.3	Salinity	1.7–2.8/Up to 5% in vitro	TAS [76]
MIGS-22	Oxygen requirement	Aerobic/Tolerate low pressure of O_2_	TAS [76, 77]
MIGS-15	Biotic relationship	free-living	IDA
MIGS-14	Pathogenicity	Pathogenic	TAS [22]
MIGS-4	Geographic location	Bogotá, Colombia	IDA
MIGS-5	Sample collection	20 May 2008	IDA
MIGS-4.1	Latitude	4.600659	IDA
MIGS-4.2	Longitude	−74.065592	IDA
MIGS-4.4	Altitude	2658 m	IDA

^a^Evidence codes: *IDA* inferred from direct assay (first time in publication); *TAS* traceable author statement (i.e., a direct report exists in the literature); *NAS* non-traceable author statement (i.e., not directly observed for the living, isolated sample, but based on a generally accepted property for the species, or anecdotal evidence). These codes are from the Gene Ontology project [56]

## Genome sequencing information

### Genome project history

The Genome of the isolate HDO1 was sequenced by NovoGene Technology Bioinformatics Co., Ltd. (Hong Kong). The whole genome shotgun project of *S. apiospermum* has been deposited in NCBI database under the accession number MVOQ00000000, belonging to the bioproject PRJNA357602. A summary of the project and information about genome sequence are shown in Table 2.

**Table 2:** Project information

MIGS ID	Property	Term
MIGS 31	Finishing quality	Draft
MIGS-28	Libraries used	One 250 pb Paired-End library, one 5 kb Mate pair library
MIGS 29	Sequencing platforms	Illumina HiSeq2500
MIGS 31.2	Fold coverage	540 x
MIGS 30	Assemblers	Abyss 1.9.0.20
MIGS 32	Gene calling method	Augustus 3.0.3
	Locus Tag	BTW05
	Genbank ID	MVOQ00000000
	GenBank Date of Release	May 26, 2017
	GOLD ID	NA
	BIOPROJECT	PRJNA357602
MIGS 13	Source Material Identifier	Strain HDO1 Museo de Historia Natural Andes
	Project relevance	Biotechnological

### Growth conditions and genomic DNA preparation

Fungus growth was carried out in liquid culture (YPG: 1% yeast extract, peptone 1% and 2% glucose) at 30 °C for 7 days, followed by vacuum filtration, lyophilization, and maceration to have a homogeneous sample. DNA was extracted by the CTAB and Phenol/Chloroform/Isoamyl alcohol method [25]. DNA quality was analyzed by Nanodrop2000 (Thermo Fisher Scientific, MA, USA) and agarose gel electrophoresis (0.8%). DNA quantity was determined by Qubit2.0 (Invitrogen, CA, USA).

### Genome sequencing and assembly

Genome sequencing of the strain was performed using high-throughput Illumina technology on a Hiseq2500 and employing two libraries: a 250 bp paired-end library and a 5kpb mate-pair library. Quality trimming of reads was performed using Trimmomactic 0.23 [35] and quality control was performed using FastQC 0.11.2 [36]. Coverage and depth of sequencing was analyzed by mapping the reads using Bowtie2–2.2.4 [37], the sam files were converted to bam files for visualization using samtools1.1 [38], and the visualization was made using tablet. 1.15.09.1 [39]. The genome was *the novo* assembled using Abyss 1.0.9.20 [40] with a kmer size of 64, scaffolds were generated with SSPACE BASIC 2.0 [41], and gaps were reduced using GapFiller 1.1 [42]. Assembly statistics were obtained using Quast 2.3 (Additional file 1: Table S1) [43]. Repetitive elements were identified with RepeatMasker 4.0.5 [44]. The draft genome of *S. apiospermum* strain HDO1 was assembled from a total of 97,208,043 reads using Abyss [40] assembler. The assembly yielded 178 scaffolds (larger than 500 bp) with a genome size of 44.2 Mbp and a G + C content of 49.91% with a mean depth of 541X. The genome assembly statistics are shown in Table 3. The total number of non-coding repetitions was found using RepeatMasker [44] and was of 1.93%. The majority of repetitions were found to be simple repeats (0.89%) and low complexity regions (0.25%). The complete report of the annotation results for the non-coding repeats sequences can be seen in the Additional file 1: Table S2. The assembly features obtained for the draft sequence were similar to other fungal genome sequence projects [23, 45, 46].

**Table 3:** Genomic statistics

Attribute	Value	% of total
Genome size (bp)	44,188,879	100
DNA coding (bp)	18,219,288	41.2
DNA G + C (bp)	22,057,939	49.91
DNA scaffolds	178	100
Total genes	11,278	100
Protein coding genes	11,184	99.16
RNA genes	92	0.81
Pseudo genes	2	0,1 × 10^−1^
Genes in internal clusters	ND	ND
Genes with function prediction	8575	76.0
Genes assigned to COGs	4789	42.4
Genes with Pfam domains	7978	70.8
Genes with signal peptides	1333	11.8
Genes with transmembrane helices	2293	20.3

### Genome annotation

**Fig. 3:**
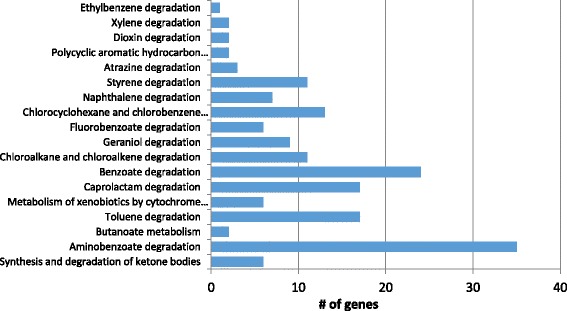
Distribution of the hydrocarbon degradation genes in KEEG pathways. The bars represent the number of genes mapped in KEEG pathways related to hydrocarbon degradation. Most of the genes were mapped to the benzoate and its derivate compounds as aminobenzoate and fluorobenzoate

Gene prediction and structure annotation was conducted using Augustus 3.0.3 [47]. Functional annotation was performed using Blast2GO 3.1 [48]. Briefly, a BLASTx against the National Center for Biotechnology Information “nr” database [49] was conducted. Then, results were classified among Gene Ontology categories [50]. Protein classification was made using the COG [51], KOG (Eukaryotic Orthologous Groups) [52] and EggNOG [53] databases using Blast2GO v4.0 platform [48]. Annotated genes were mapped against Kyoto encyclopedia of genes and genomes [54] to its functional analysis and assigned the Enzyme Codes. A total of 11,195 protein-encoding genes were predicted using Augustus [47]. Functional annotation showed a total of 8595 (76.0% of predicted genes) sequences with predicted function using Blastx [49]. Then, InterProScan [55] and Gene Ontology [56] permitted the annotation of 7934 (70.3%) sequences with GO terms, whilst the remaining genes were annotated as hypothetical (17.1%) and unknown function proteins (5.0%). A total of 7978 (70.8%) genes contained pfam [57] domains and 1333 had signal peptide domains. The transmembrane helices in the proteins were predicted with TMHMM sever v.2.0 in the online portal [58]. The ribosomal RNA genes were predicted in the RNAmmer 1.2 Server [59] and making an alignment with the predicted genes for *Neurospora crassa* from the database FungiDB [60], same database was used for pseudogenes prediction comparing with pseudogenes predicted for *Neurospora crassa*. The statistics of the genome annotation are shown in Table 3. A total of 4789 (42.5%) genes were assigned to the KOG [61] categories, most of them (60%) were assigned to one or more functional groups and the rest of genes were assigned to the function unknown group (Table 4). KEGG pathway analysis assigned an enzyme code to 2645 (23.5%) genes and revealed specific genes involved in the pathways of hydrocarbon degradation. These hydrocarbons are chloroalkane/alkene, chlorocyclohexane and chlorobenzene, benzoate, aminobenzoate, fluorobenzoate, toluene, caprolactam, geraniol, naphthalene, styrene, atrazine, dioxin, xylene, ethylbenzene, and polycyclic aromatic hydrocarbons. Also, the analysis revealed the presence of genes involved in metabolism of xenobiotics by cytochrome P450 and in synthesis and degradation of ketone bodies. These results are shown in Fig. 3.

**Table 4:** Number of genes associated with general COG functional categories

Code	Value	% age	Description
J	234	2.09	Translation, ribosomal structure and biogenesis
A	42	0.37	RNA processing and modification
K	163	1.46	Transcription
L	126	1.12	Replication, recombination and repair
B	19	0.17	Chromatin structure and dynamics
D	32	0.29	Cell cycle control, Cell division, chromosome partitioning
V	32	0.29	Defense mechanisms
T	153	1.37	Signal transduction mechanisms
M	126	0.44	Cell wall/membrane biogenesis
N	2	0.02	Cell motility
U	241	2.15	Intracellular trafficking and secretion
O	294	2.63	Posttranslational modification, protein turnover, chaperones
C	216	1.93	Energy production and conversion
G	399	3.56	Carbohydrate transport and metabolism
E	257	2.30	Amino acid transport and metabolism
F	65	0.58	Nucleotide transport and metabolism
H	63	0.56	Coenzyme transport and metabolism
I	140	1.25	Lipid transport and metabolism
P	131	1.17	Inorganic ion transport and metabolism
Q	186	1.66	Secondary metabolites biosynthesis, transport and catabolism
R	0	0	General function prediction only
S	1918	17.14	Function unknown
–	6402	57.2	Not in KOGs

## Genome properties

The assembled genome of the strain HDO1 has a size of 44,188,879 pb (distributed in 178 scaffolds) with a G-C content of 49.91%; the genome size and the G-C content was similar to the draft genome reported for the strain IHEM 14462 [22] (Table 5). A total of 11,278 genes were predicted; among these, 11,184 were identified as coding protein genes (representing the 99.16% of the total genes); 92 as RNA genes (0.81%); and 2 as pseudogenes (0.02%) (Table 3). Some other features of the predicted genes are shown in Table 4. The number of chromosomes could not be elucidated.

**Table 5:** Genomic features comparison between HDO1 strain and IHEM 14462 strain [22]

Parameter	IHEM 14462	HDO1
Size (Mb)	43.44	44.19
Content G-C (%)	50.4	49.91
Predicted genes	10,919	11,278
Predicted proteins	8.375	11,184

## Insights from the genomic sequence

### Comparative genomics

**Fig. 4:**
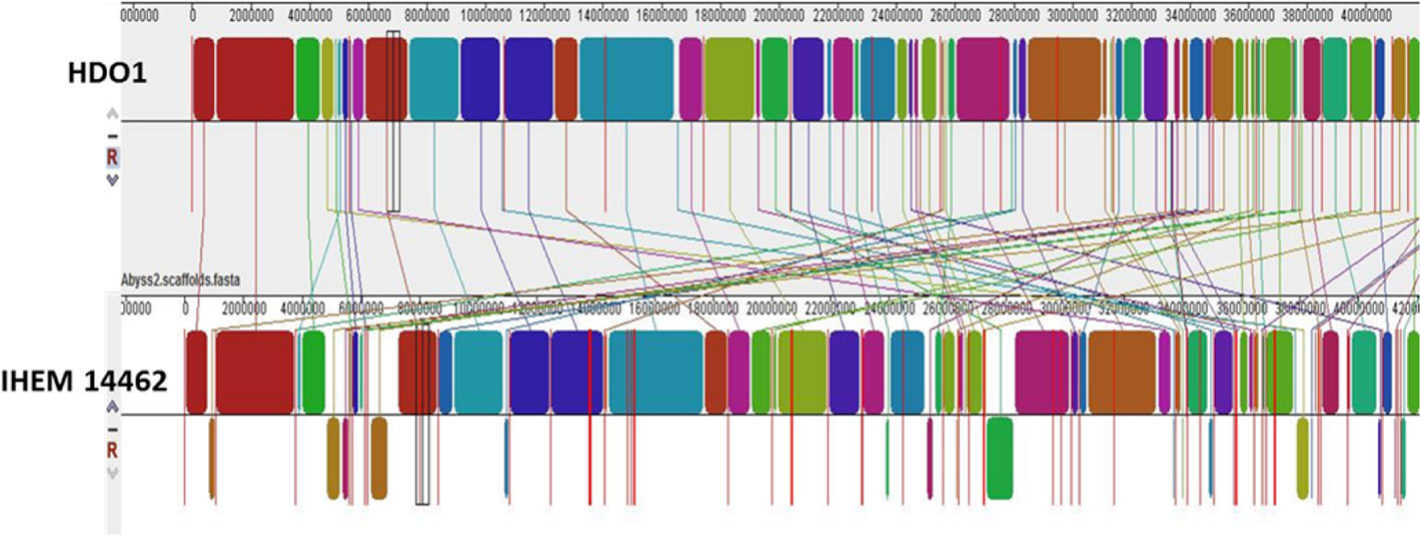
MAUVE [62] alignment of draft genome sequence of HDO1 strain and draft genome sequence of IHEM 14462 strain. The figure represents the locally contiguous blocks (LCBs) that both sequences share, connected by lines to show their positions in the genomes. At the top the sequence of HDO1 strain is visualized and at the bottom the re-ordered sequence of the IHEM 14462 strain appears [23]. Blocks that are shown below indicate regions that have the reverse sequence related to the HDO1 sequence

**Fig. 5:**
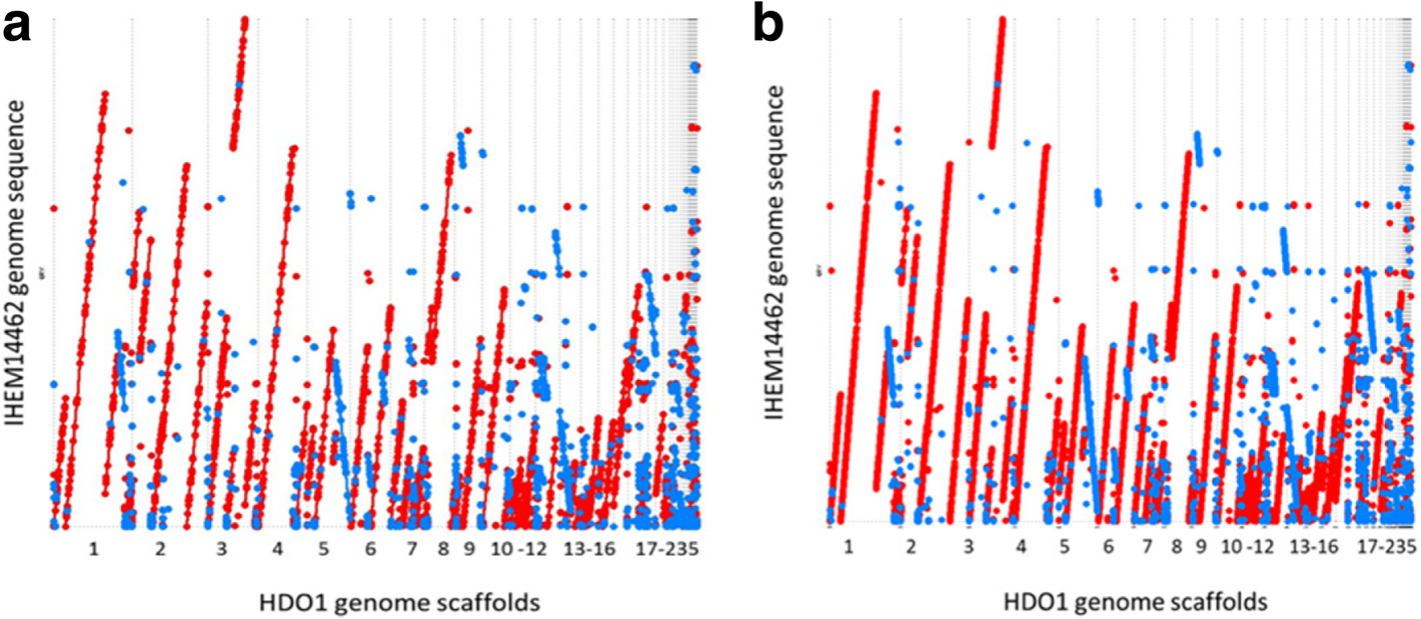
Dot plot analysis comparing the HDO1 and IHEM14462 strains’ genomes. **a** Comparison at the nucleotide level. **b** Comparison at the protein level. It shows the alignment of the genome sequence of IHEM 14462 strains (y axis) against HDO1 genome sequence (x axis). The red color lines and dots represent the forward matches between the both genome sequences while the blue color ones represent reverse matches

Reads were mapped versus the clinical strain 10.1601/strainfinder?urlappend=%3Fid%3DIHEM+14462 using Bowtie2–2.2.4 [26]. The sam files were converted to bam files for the visualization using samtools1.1 [27] and the visualization was made using tablet. 1.15.09.1 [28] resulting in an overall alignment of 92.75%. Genomes’ comparison between the environmental strain HDO1 and the clinical strain 10.1601/strainfinder?urlappend=%3Fid%3DIHEM+14462 was performed using MAUVE 20150226 [62]. The genome sequence of HDO1 strain aligned with the sequence of 10.1601/strainfinder?urlappend=%3Fid%3DIHEM+14462 strain in 88,1% of its length. The MAUVE [62] alignment showed a high level of similarity between the clinical and the environmental strains (Fig. 4). A total of 508 local collinear blocks (LCBs) that correspond to the homologous regions that are shared by the two sequences were found and a few of them were in reverse orientation after eight reordered cycles. From ordered output fasta file obtained with MAUVE [48] a new alignment was made with Nucmer at nucleotide level (maximum gap between two adjacent matches in a cluster of 90 bp and a minimum length of a maximal exact match of 20 bp) and Promer at amino acid level (maximum gap between two adjacent matches in a cluster of 30 amino acids and a minimum length of a maximal exact match of 6 amino acids). Nucmer and Promer alignments were plotted using Mumerplot, the last three mentioned tools from mummer 3.0 suite [63] (Fig. 5). This analysis revealed that a high number of forward matches are in the greatest scaffolds of HDO1 genome sequence and reverse matches are more common in the smallest scaffolds. These differences and similarities seen for the nucleotides showed the same trends when these were translated to amino acids. These analyses and their corresponding plots also permitted to determine rearrangements, insertions, and deletions between both genomes.

A thorough comparative analysis showed some important differences between the genome draft sequences of the clinical and the environmental strain sequenced here. These differences were evident in the genome size of the assemblies and the number of predicted genes (Table 5). Indeed, our assembly had a total of 783.135 bp (1.77% of genome size) and 276 coding sequences more than the clinical strain. The remarkable difference in the number of annotated genes involved in hydrocarbons degradation pathways could be attributed to the pipeline followed to annotate genes. For the clinical strain the CDSs found were annotated against TrEmbl database [64] that only comprises UniProtKB/Swiss-Prot, while in this study, we used the nr (non-redundant protein sequences) database of NCBI which has a wider coverage because it comprises sequences obtained from another databases like GenPept, TPA, PIR, PRF, PDB, NCBI RefSeq, and UniProtKB/Swiss-Prot [65]. Since the repetitive elements of the genome were estimated as only 1.93%, it is highly probable that the difference in size can be attributed to some of the elements involved in functional categories.

### Genes involved in hydrocarbon biodegradation pathways

Several genes involved in hydrocarbon biodegradation pathways were annotated in the genome of the environmental strain. In Table 6 the genes previously reported in the clinical strain [23] are shown. Results revealed that some genes are involved in several degradation pathways, principally corresponding to aromatic hydrocarbon metabolism (polycyclic aromatic hydrocarbons and phenolic compounds) and cytochrome P450 system. The number of these genes annotated for each strain can also be seen in the table and these values showed a higher number of genes in the environmental strain HDO1. The genes solely found in the draft sequence of the strain HDO1 are reported in Table 7. These genes comprised some genes belonging to the aromatic hydrocarbons degradation pathways completing the pathways in which genes found in both strains are also involved. Genes involved in the degradation of other organic compounds like toluene, lignin, and xylenol were found (Table 6).

**Table 6:** Annotated genes involved in hydrocarbons degradation pathways

Gene	Pathway	# of genes in HDO1	# of genes in IHEM 14462 [23]
Cytochrome P450 monooxygenase (EC:1.14.13.12)	PAHs degradation, alkane biodegradation [78, 79]	79	44
Phenol hydroxylase	Phenol degradation [17]	4	4
Epoxide hydrolase (EC:3.3.2.9)	PAHs degradation [80]	3	2
Oxidoreductase	Organic compound metabolism [81]	13	8
Salicylate hydroxylase (EC:1.14.13.24)	Naphthalene degradation [82]	13	4
Laccase (EC:1.10.3.2)	PAHs degradation [83]	2	2
Catechol 1,2-dioxygenase (EC:1.13.11.1)	Phenol degradation [17]		
2,4-dichlorophenol 6-monooxygenase (EC:1.14.13.7; EC:1.14.13.20)	Chlorinated phenols degradation [84]	5	5
2,3-dihydroxybenzoate decarboxylase (EC:4.1.1.46)	2,3-dihydroxybenzoate degradation [85]	1	1
Carboxy-cis,cis-muconate cyclase	Phenol degradation [17]	4	2
Phenylacetate 2-hydroxylase	Homogentisate degradation [86]	1	2
2-nitropropane dioxygenase (EC:1.13.12.16)	Nitroalquene oxidation [87]	4	4
Biphenyl-2,3-diol 1,2-dioxygenase	Byphenyl degradation [88]	1	2
Dienelactone hydrolase	Chloroaromatic degradation [89]	2	7
Vanillyl-alcohol oxidase (EC:1.1.3.38)	Aromatic degradation [90]	4	6
Cyclopentanone 1,2-monooxygenase (EC:1.14.13.8; EC:1.14.13.16)	Cyclopentanol degradation [91]	2	2
Tyrosinase	Phenolic compounds degradation [92]	1	3
Lignostilbene dioxygenase (EC:1.13.11.43)	Lignin degradation [93]	2	1
Total number of genes		145	103

**Table 7:** Annotated genes found only in the HDO1 strain

Genes in HDO1	Pathway	# of genes
3-oxoadipate enol-lactonase	Phenol degradation [94]	5
5-carboxymethyl-2-hydroxymuconate isomerase	Homoprotocatechuate degradation pathway [95]	1
Trihydroxytoluene oxygenase	2,4-dinitrotoluene degradation [96]	1
Benzoate 4-monooxygenase (EC:3.6.1.3)	Benzoate degradation [18]	5
Diphenol oxidase	Phenolic compounds degradation [97]	1
Cyclohexanone monooxygenase (EC:1.14.13.8)	Cyclohexane degradation [98]	4
Gentisate 1,2-dioxygenase	PAHs degradation [99]	2
2-keto-4-pentenoate hydratase (EC:3.7.1.5)	Benzoate degradation [100]	1
carboxymuconolactone decarboxylase	Protocatechuate degradation [101]	1
3-(3-hydroxy-phenyl)propionate 3-hydroxycinnamic acid hydroxylase	Phenyl propionate degradation [102]	2
3-hydroxybenzoate 6-hydroxylase	Xylenol [103] and 3-hydroxybenzoate degradation [104]	1
3-hydroxyisobutyrate dehydrogenase (EC:1.1.1.44; EC:2.1.1.43)	Aromatic compounds metabolism [105]	5
Total number of genes		30

The complete annotation of the genome and, particularly, of the genes belonging to a major class of protein families involved in fungal catabolism of organic pollutants was made. We could identify genes coding for proteins that have the ability to oxidize aromatic compounds like dioxygenases or monooxygenases. Among these, we could predict dioxygenases such as 2-nitropropane dioxygenase, extracellular dioxygenase (EC:1.13.11), gentisate 1,2-dioxygenase, intradiol ring-cleavage dioxygenase (EC:1.13.11), lignostilbene dioxygenase (EC:1.13.11.43), catechol 1,2-dioxygenase (EC:1.13.11.1), biphenyl-2,3-diol 1,2-dioxygenase, aromatic ring-opening dioxygenase, and 4-hydroxyphenylpyruvate dioxygenase (EC:1.13.11.27). These enzymes have great importance because, along with NADH-dependent flavin reductase and [2Fe-2S] redox centers, they catalyze the transformation of several aromatic compounds to dihydrodiols [66], allowing the complete mineralization of these compounds to CO_2_ and H_2_O (with the participation of other specific enzymes). Another enzyme family identified among the annotated genes was cytochrome P450. These enzymes have an interesting catabolic potential because they do not have substrate specificity and can catalyze epoxidation and hydroxylation of several organic pollutants like dioxins, nonylphenol, and PAHs [67]. Genes coding for extracellular proteins like laccases and tyrosinase (known as phenoloxidase enzymes), which have the ability to degrade several groups of organic compounds due to their non-specificity action, were annotated in the genome. These enzymes produce organic radicals beyond one electron abstraction; those free radicals can be transformed by several reactions that include the ether cleavage in dioxins, quinone formations from PAHs and chlorophenols [68]. These extracellular enzymes are extremely important because of their potential in biotechnological applications [69, 70]. Moreover, several oxidoreductases, hydrolases, dehydroxylases, isomerases, and transferases were also predicted in the studied strain. However, extracellular enzymes such as lignin and manganese peroxidases could not be identified yet.

Catabolic proteins of *S. apiospermum* involved in phenol, p-cresol and phenylbenzoate degradation pathway previously reported by (Clauβen and Schmidt) [17, 18] like phenol 2-monooxygenase and cathecol 1,2 dioxygenase were identified. However, hydroquinone hydroxylase, 4-hydroxybenzoate 3-hydroxylase, hydroxiquinone 1,2 dioxygenase, protocatechuate 3,4 dioxygenase, and maleylacetate reductase could not be found, suggesting that these proteins could be classified among the proteins annotated as hypothetical or with an unknown function or that they can be in the gap regions of the genome assembly.

## Conclusions

The draft genome sequence of environmental strain *S. apiospermum* HDO1 isolated from bacterial bioremediation assays in crude oil was described here. The structural and functional information of the genome sequence of *S. apiospermum* has allowed advancing in the understanding of the ability of this fungus to degrade several kinds of xenobiotic compounds mainly several hydrocarbons families and offers an opportunity to propose its use or its enzymes in controlled bioremediation or bioaugmentation processes.

## Additional files

Additional file 1: Table S1. Genome assembly statistics reported by Quast [44]. **Table S2.** Non-coding repeats sequences summary. **Table S3.** Species and genes (accession numbers) used in the phylogenetic analysis [29]. (DOCX 37 kb)

## Abbreviations

API: American Petroleum Institute
CDS: Coding DNA Sequence
COG: Cluster of Orthologous Groups
CTAB: Cetyl Trimethyl Ammonium Bromide
EC: Enzyme Codes
GO: Gene Ontology
ITS: Internal Transcribed Spacer
KEGG: Kyoto encyclopedia of genes and genomes
KOG: Eukaryotic Orthologous Groups
LSU: long subunit rRNA
PAHs: Polycyclic Aromatic hydrocarbons
PDA: Potato Dextrose Agar
PDB: Protein Data Bank Protein
PIR: The Protein Information Resource
PRF: Protein Research Foundation
TEF: Elongation factor
TPA: Third-Party Annotation Sequences
YPG: Yeast extract, Peptone, Glucose

## References

[1] Atlas RM (1981). Microbial degradation of petroleum hydrocarbons: an environmental perspective. Microbiol Rev.

[2] Baker SE (2008). Fungal genome sequencing and bioenergy. Fungal Biol Rev.

[3] Haritash A, Kaushik C (2009). Biodegradation aspects of polycyclic aromatic hydrocarbons (PAHs): a review. J Hazard Mater.

[4] Leahy JG, Colwell RR (1990). Microbial degradation of hydrocarbons in the environment. Microbiol Rev.

[5] April TM, Foght J, Currah R (1999). Hydrocarbon-degrading filamentous fungi isolated from flare pit soils in northern and western Canada. Can J Microbiol.

[6] Semple KT, Cain RB, Schmidt B (1999). Biodegradation of aromatic compounds by microalgae. FEMS Microbiol Lett.

[7] Smith, M.R. The biodegradation of aromatic hydrocarbons by bacteria, in Physiology of Biodegradative Microorganisms. 1991, Springer. p. 191–206.

[8] Grady CL (1990). Biodegradation of toxic organics: status and potential. J Environ Eng.

[9] Samanta SK, Singh OV, Jain RK (2002). Polycyclic aromatic hydrocarbons: environmental pollution and bioremediation. Trends Biotechnol.

[10] Providenti MA, Lee H, Trevors JT (1993). Selected factors limiting the microbial degradation of recalcitrant compounds. J Ind Microbiol.

[11] Morgan P, Atlas RM (1989). Hydrocarbon degradation in soils and methods for soil biotreatment. Crit Rev Biotechnol.

[12] Hickey WJ, Chen S, Zhao J (2012). The phn island: a new genomic island encoding catabolism of polynuclear aromatic hydrocarbons. Front Microbiol.

[13] Hickey WJ (2011). Development of tools for genetic analysis of phenanthrene degradation and nanopod production by Delftia sp. Cs1-4. Front Microbiol.

[14] Desai C, Pathak H, Madamwar D (2010). Advances in molecular and “-omics” technologies to gauge microbial communities and bioremediation at xenobiotic/anthropogen contaminated sites. Bioresour Technol.

[15] Gilgado F, Gené J, Cano J, Guarro J (2010). Heterothallism in Scedosporium apiospermum and description of its teleomorph Pseudallescheria apiosperma sp. nov. Med Mycol.

[16] Kaltseis J, Rainer J, De Hoog GS (2009). Ecology of Pseudallescheria and Scedosporium species in human-dominated and natural environments and their distribution in clinical samples. Med Mycol.

[17] Claußen M, Schmidt S (1998). Biodegradation of phenol and p-cresol by the hyphomycete Scedosporium apiospermum. Res Microbiol.

[18] Clauβen M, Schmidt S (1999). Biodegradation of phenylbenzoate and some of its derivatives by Scedosporium apiospermum. Res Microbiol.

[19] García-Peña EI, Hernández S, Favela-Torres E, Auria R, Revah S (2001). Toluene biofiltration by the fungus Scedosporium apiospermum TB1. Biotechnol Bioeng.

[20] Reyes-César A, Absalón A, Fernández F, González J, Cortés-Espinosa (2014). Biodegradation of a mixture of PAHs by non-ligninolytic fungal strains isolated from crude oil-contaminated soil. World J Microbiol Biotechnol.

[21] Sandoval, C. Pijarán, J. C. Vives-Florez, M. Remoción de hidrocarburos por hongos filamentosos. Universidad de los Andes (Master's Thesis ), 2012.

[22] Guarro J, Kantarcioglu A, Horré R, Rodriguez-Tudela J, Cuenca M, Berenguer J, De Hoog G (2006). Scedosporium apiospermum: changing clinical spectrum of a therapy-refractory opportunist. Med Mycol.

[23] Vandeputte P (2014). Draft genome sequence of the pathogenic fungus Scedosporium apiospermum. Genome Announcements.

[24] Küppers, H., Atlas de los colores. 3ͣ Edición. Barcelona, Blume, S.A., 1996(ISBN 84–87535–37-2.).

[25] Vasco MF, Cepero M, Restrepo S, Vives-Florez M (2011). Recovery of mitosporic fungi actively growing in soils after bacterial bioremediation of oily sludge and their potential for removing recalcitrant hydrocarbons. Int Biodeterior Biodegrad.

[26] White TJ, Bruns T, Lee D, Taylor SB, J. W. (1990). Amplification and direct sequencing of fungal ribosomal RNA genes for phylogenetics. PCR Protocols.

[27] Sandoval-Denis M, Sutton DA, Fothergill AW, Cano-Lira JF, Gené J, Decock C, De Hood GS, Guarro J (2013). Scopulariopsis, a poorly known opportunistic fungus: spectrum of species in clinical samples and in vitro responses to antifungal drugs. J Clin Microbiol.

[28] Sandoval-Denis M (2016). Phylogeny and taxonomic revision of Microascaceae with emphasis on synnematous fungi. Stud Mycol.

[29] Katoh K, Toh H (2010). Parallelization of the MAFFT multiple sequence alignment program. Bioinformatics.

[30] Stamatakis A (2006). RAxML-VI-HPC: maximum likelihood-based phylogenetic analyses with thousands of taxa and mixed models. Bioinformatics.

[31] Miller, M.A. Pfeiffer, W. Schwartz, T. Creating the CIPRES Science Gateway for inference of large phylogenetic trees. in Gateway Computing Environments Workshop (GCE), 2010. 2010. IEEE.

[32] Lanfear R, Calcott B, Ho SYW, Guindon S (2012). PartitionFinder: combined selection of partitioning schemes and substitution models for phylogenetic analyses. Mol Biol Evol.

[33] Stamatakis A (2014). RAxML version 8: a tool for phylogenetic analysis and post-analysis of large phylogenies. Bioinformatics.

[34] Rambaut, A. FigTree, a graphical viewer of phylogenetic trees. See http://tree.bio.ed.ac.uk/software/figtree, 2007.

[35] Bolger, A.M. Lohse, M. Usadel, B. Trimmomatic: a flexible trimmer for Illumina sequence data. Bioinformatics, 2014: p. btu170. 10.1093/bioinformatics/btu170 PMC4103590 24695404

[36] Andrews, S.F. and Q. Fast, A quality control tool for high throughput sequence data. 2010, 2015.

[37] Langmead B, Salzberg SL (2012). Fast gapped-read alignment with bowtie 2. Nat Methods.

[38] Li H (2009). The sequence alignment/map format and SAMtools. Bioinformatics.

[39] Milne I, Bayer M, Cardle L, Shaw P, Stephen G, Wright F, Marshall D (2010). Tablet—next generation sequence assembly visualization. Bioinformatics.

[40] Simpson JT, Wong K, Jackman S, Schein D, Jones JE, Birol SJ, I. (2009). ABySS: a parallel assembler for short read sequence data. Genome Res.

[41] Boetzer M, Henkel C, Jansen V, Butler HJ, Pirovano D, W. (2011). Scaffolding pre-assembled contigs using SSPACE. Bioinformatics.

[42] Boetzer M, Pirovano W (2012). Toward almost closed genomes with GapFiller. Genome Biol.

[43] Gurevich A, Saveliev V, Vyahhi N, Tesler G (2013). QUAST: quality assessment tool for genome assemblies. Bioinformatics.

[44] Smit, A.F. Hubley, R. Green, P. *RepeatMasker.* Published on the web at http://www. repeatmasker.org, 1996.

[45] Yew SM (2016). The genome of newly classified Ochroconis mirabilis: insights into fungal adaptation to different living conditions. BMC Genomics.

[46] Galagan JE (2003). The genome sequence of the filamentous fungus Neurospora crassa. Nature.

[47] Stanke M, Morgenstern B (2004). AUGUSTUS: a web server for gene finding in eukaryotes. Nucleic Acids Res.

[48] Conesa A, Götz S, García-Gómez JM, Terol J, Talón M, Robles M (2005). Blast2GO: a universal tool for annotation, visualization and analysis in functional genomics research. Bioinformatics.

[49] Altschul SF, Gish W, Miller W, Myers EW, Lipman DJ (1990). Basic local alignment search tool. J Mol Biol.

[50] Consortium GO (2004). The gene ontology (GO) database and informatics resource. Nucleic Acids Res.

[51] Tatusov RL, Galperin MY, Natale DA, Koonin EV (2000). The COG database: a tool for genome-scale analysis of protein functions and evolution. Nucleic Acids Res.

[52] Koonin EV (2004). A comprehensive evolutionary classification of proteins encoded in complete eukaryotic genomes. Genome Biol.

[53] Powell S (2012). eggNOG v3. 0: orthologous groups covering 1133 organisms at 41 different taxonomic ranges. Nucleic Acids Res.

[54] Kanehisa M, Goto S (2000). KEGG: kyoto encyclopedia of genes and genomes. Nucleic Acids Res.

[55] Zdobnov EM, Apweiler R (2001). InterProScan–an integration platform for the signature-recognition methods in InterPro. Bioinformatics.

[56] Ashburner M (2000). Gene ontology: tool for the unification of biology. Nat Genet.

[57] Sonnhammer EL, Eddy SR, Birney E, Bateman A, Durbin R (1998). Pfam: multiple sequence alignments and HMM-profiles of protein domains. Nucleic Acids Res.

[58] Krogh A, Larsson B, von Heijne G, Sonnhammer EL (2001). Predicting transmembrane protein topology with a hidden Markov model: application to complete genomes. J Mol Biol.

[59] Lagesen K, Hallin P, Rodland EA, Staerfieldt HH, Rognes T, Ussery DW (2007). RNammer: consistent annotation of rRNA genes in genomic sequences. Nucleic Acids Res.

[60] Stajich JE (2012). FungiDB: an integrated functional genomics database for fungi. Nucleic Acids Res.

[61] Yong Jung W, Sook Lee S, Wook Kim C, Kim, H. S, Ran Min S, Moon J. S, Kwon S.Y, Jeon J. H, Sun Cho H. Eukaryotic Orthologous Groups (KOG) classification of the assembled loci. figshare., 2014.

[62] Darling AE, Mau B, Perna NT (2010). progressiveMauve: multiple genome alignment with gene gain, loss and rearrangement. PloS one.

[63] Kurtz S, Phillippy A, Delcher AL, Smoot M, Shumway M, Antonescu C, Salzberg SL (2004). Versatile and open software for comparing large genomes. Genome Biol.

[64] Magrane M, Consortium U (2011). UniProt knowledgebase: a hub of integrated protein data. Database.

[65] Apweiler R (2004). UniProt: the universal protein knowledgebase. Nucleic Acids Res.

[66] Mason JR, Cammack R (1992). The electron-transport proteins of hydroxylating bacterial dioxygenases. Annu Rev Microbiol.

[67] Kasai N, Ikushiro SI, Shinkyo R, Yasuda K, Hirosue S, Arisawa A, Ichinose H, Wariishi H, Sakaki T (2010). Metabolism of mono-and dichloro-dibenzo-p-dioxins by Phanerochaete chrysosporium cytochromes P450. Appl Microbiol Biotechnol.

[68] Harms H, Schlosser D, Wick LY (2011). Untapped potential: exploiting fungi in bioremediation of hazardous chemicals. Nat Rev Microbiol.

[69] Duran N, Esposito E (2000). Potential applications of oxidative enzymes and phenoloxidase-like compounds in wastewater and soil treatment: a review. Appl Catal B Environ.

[70] Gianfreda L, Rao MA (2004). Potential of extra cellular enzymes in remediation of polluted soils: a review. Enzym Microb Technol.

[71] Hibbett DS (2007). A higher-level phylogenetic classification of the fungi. Mycol Res.

[72] Gilgado F, Cano J, Gené J, Sutton DA, Guarro J (2008). Molecular and phenotypic data supporting distinct species statuses for Scedosporium apiospermum and Pseudallescheria boydii and the proposed new species Scedosporium dehoogii. J Clin Microbiol.

[73] Castellani, A. Chambers, A. J. Manual of tropical medicine. 1919: William Wood.

[74] Delhaes, L., Molecular Typing of Australian Scedosporium Isolates Showing Genetic Variability and Numerous S. aurantiacum. Risk. 8: p. 34.8. 10.3201/eid1402.070920 PMC2600218 18258122

[75] Dabrowa N, Landau JW, Newcomer VD, Plunkett OA (1964). A survey of tide-washed coastal areas of southern California for fungi potentially pathogenic to man. Mycopathologia.

[76] Hoog Gd, Marvin-Sikkema FD, Lahpoor GA, Gottschall JC, Prins RA, Guého E (1994). Ecology and physiology of the emerging opportunistic fungi Pseudallescheria boydii and Scedosporium prolificans. Mycoses.

[77] Eggertsberger M, Rainer J, Kaltseis J, Poeder, R. Temperature dependence and environmental conditions enhancing the distribution of the opportunistic pathogens P. boydii and S. apiospermum. in MYCOSES. 2008. Wiley-Blackwell commerce place, 350 main St, MALDEN 02148, MA USA.

[78] Martinez D (2004). Genome sequence of the lignocellulose degrading fungus Phanerochaete chrysosporium strain RP78. Nat Biotechnol.

[79] Vatsyayan P, Kamur AK, Goswami P, Goswami P (2008). Broad substrate cytochrome P450 monooxygenase activity in the cells of Aspergillus terreus MTCC 6324. Bioresour Technol.

[80] Cerniglia CE, Hebert RL, Szaniszlo PJ, Gibson DT (1978). Fungal transformation of naphthalene. Arch Microbiol.

[81] Asgher M, Bhatti HN, Ashraf M, Legge RL (2008). Recent developments in biodegradation of industrial pollutants by white rot fungi and their enzyme system. Biodegradation.

[82] You IS, Ghosal D, Gunsalus IC (1991). Nucleotide sequence analysis of the pseudomonas putida PpG7 salicylate hydroxylase gene (nahG) and its 3′-flanking region. Biochemistry.

[83] Novotný Č, Svobodová K, Erbanová P, Cajthaml T, Kasinath A, Lang E, Sasek V (2004). Ligninolytic fungi in bioremediation: extracellular enzyme production and degradation rate. Soil Biol Biochem.

[84] Bollag J, Helling C, Alexander M (1968). 2, 4-D metabolism. Enzymic hydroxylation of chlorinated phenols. J Agric Food Chem.

[85] Anderson JJ, Dagley S (1981). Catabolism of tryptophan, anthranilate, and 2, 3-dihydroxybenzoate in Trichosporon cutaneum. J Bacteriol.

[86] Mingot JM, Peñalva MA, Fernández-Cañón JM (1999). Disruption of phacA, an Aspergillus nidulans gene encoding a novel cytochrome P450 monooxygenase catalyzing phenylacetate 2-hydroxylation, results in penicillin overproduction. J Biol Chem.

[87] Kido T, Soda K (1984). Oxidation of anionic nitroalkanes by flavoenzymes, and participation of superoxide anion in the catalysis. Arch Biochem Biophys.

[88] Yang X, Xie F, Zhang G, Shi Y, Qian S (2008). Purification, characterization, and substrate specificity of two 2, 3-dihydroxybiphenyl 1, 2-dioxygenase from Rhodococcus sp. R04, showing their distinct stability at various temperature. Biochimie.

[89] Schlömann M, Schmidt E, Knackmuss H (1990). Different types of dienelactone hydrolase in 4-fluorobenzoate-utilizing bacteria. J Bacteriol.

[90] Fraaije MW, Pikkemaat M, Van Berkel W (1997). Enigmatic gratuitous induction of the covalent Flavoprotein Vanillyl-alcohol Oxidase in Penicillium simplicissimum. Appl Environ Microbiol.

[91] Iwaki H, Hasegawa Y, Wang S, Kayser MM, Lau PC (2002). Cloning and characterization of a gene cluster involved in cyclopentanol metabolism in Comamonas sp. strain NCIMB 9872 and biotransformations effected by Escherichia Coli-expressed cyclopentanone 1, 2-monooxygenase. Appl Environ Microbiol.

[92] Ikehata K, Nicell JA (2000). Characterization of tyrosinase for the treatment of aqueous phenols. Bioresour Technol.

[93] Kamoda S, Habu N, Samejima M, Yoshimoto T (1989). Purification and some properties of Lignostilbene-a,/i-dioxygenase responsible for the ca—cp cleavage of a Diarylpropane type lignin model compound from pseudomonas sp. TMY1009. Agric Biol Chem.

[94] Harwood CS, Parales RE (1996). The β-ketoadipate pathway and the biology of self-identity. Annu Rev Microbiol.

[95] Roper DI, Cooper RA (1990). Purification, some properties and nucleotide sequence of 5-carboxymethyl-2-hydroxymuconate isomerase of Escherichia Coli C. FEBS Lett.

[96] Johnson GR, Jain RK, Spain JC (2000). Properties of the trihydroxytoluene oxygenase from Burkholderia cepacia R34: an extradiol dioxygenase from the 2, 4-dinitrotoluene pathway. Arch Microbiol.

[97] Williamson PR (1994). Biochemical and molecular characterization of the diphenol oxidase of Cryptococcus Neoformans: identification as a laccase. J Bacteriol.

[98] Sheng D, Ballou DP, Massey V (2001). Mechanistic studies of cyclohexanone monooxygenase: chemical properties of intermediates involved in catalysis. Biochemistry.

[99] Luo S, Liu DQ, Liu H, Zhou NY (2006). Site-directed mutagenesis of gentisate 1, 2-dioxygenases from Klebsiella Pneumoniae M5a1 and Ralstonia sp. strain U2. Microbiol Res.

[100] Perez-Pantoja D, De la Iglesia R, Pieper DH, González B (2008). Metabolic reconstruction of aromatic compounds degradation from the genome of the amazing pollutant-degrading bacterium Cupriavidus necator JMP134. FEMS Microbiol Rev.

[101] Eulberg D, Lakner S, Golovleva LA, Schlömann M (1998). Characterization of a protocatechuate catabolic gene cluster from Rhodococcus opacus 1CP: evidence for a merged enzyme with 4-carboxymuconolactone-decarboxylating and 3-oxoadipate enol-lactone-hydrolyzing activity. J Bacteriol.

[102] Burlingame R, Chapman PJ (1983). Catabolism of phenylpropionic acid and its 3-hydroxy derivative by Escherichia Coli. J Bacteriol.

[103] Poh CL, Bayly RC (1980). Evidence for isofunctional enzymes used in m-cresol and 2, 5-xylenol degradation via the gentisate pathway in Pseudomonas Alcaligenes. J Bacteriol.

[104] Park M, Jeon Y, Ho Hee J, Hyun-su R, Woodjun P, Madsen EL, Che Ok J (2007). Molecular and biochemical characterization of 3-hydroxybenzoate 6-hydroxylase from Polaromonas naphthalenivorans CJ2. Appl Environ Microbiol.

[105] Masai E, Katayama Y, Fukuda M (2007). Genetic and biochemical investigations on bacterial catabolic pathways for lignin-derived aromatic compounds. Biosci Biotechnol Biochem.

