# Supplementary material for: The genomic study of an environmental isolate of Scedosporium apiospermum shows its metabolic potential to degrade hydrocarbons
Source: Stand Genomic Sci. 2017 Dec 4;12:71. doi: 10.1186/s40793-017-0287-6 (PMC5716253; doi:10.1186/s40793-017-0287-6)
Supplement: Supplementary file 1 — Genome assembly statistics reported by Quast [44]. Table S2. Non-coding repeats sequences summary. Table S3. Species and genes (accession numbers) used in the phylogenetic analysis [29]. (DOCX 37 kb) [file 40793_2017_287_MOESM1_ESM.docx]

**Additional file 1**

**Table S1.** Genome assembly statistics reported by Quast [[44](#_ENREF_44)].

| **Attribute** | **value** |
| --- | --- |
| Genome size (pb) | 44,188,879 |
| DNA contigs | 2,254 |
| DNA Scaffolds | 178 |
| Largest contig | 5,361,369 |
| GC (%) | 49.91 |
| N50 | 2,801,028 |
| N75 | 1,627,699 |
| L50 | 6 |
| L75 | 12 |

**Table S2.** Non-coding repeats sequences summary.

| **Element** | **Feature** | **Size (bp)** | **%** |
| --- | --- | --- | --- |
| Retroelements | 625 | 116890 | 0.16 |
| SINEs: | 0 | 0 | 0.00 |
| [Penelope](http://www.ncbi.nlm.nih.gov/Taxonomy/Browser/wwwtax.cgi?lvl=0&id=8985) | 0 | 0 | 0.00 |
| LINEs: | 17 | 1563 | 0.00 |
| CRE/SLACS | 0 | 0 | 0.00 |
| L2/CR1/Rex | 0 | 0 | 0.00 |
| R1/LOA/Jockey | 0 | 0 | 0.00 |
| R2/R4/NeSL | 0 | 0 | 0.00 |
| RTE/Bov-B | 0 | 0 | 0.00 |
| L1/CIN4 | 0 | 0 | 0.00 |
| LTR elements: | 608 | 115327 | 0.15 |
| BEL/[Pao](http://www.ncbi.nlm.nih.gov/Taxonomy/Browser/wwwtax.cgi?lvl=0&id=1820602) | 0 | 0 | 0.00 |
| Ty1/Copia | 147 | 23725 | 0.05 |
| Gypsy/DIRS1 | 459 | 91432 | 0.19 |
| Retroviral | 0 | 0 | 0.00 |
| DNA transposons | 54 | 6910 | 0.01 |
| hobo-Activator | 5 | 413 | 0.00 |
| Tc1-IS630-Pogo | 14 | 2674 | 0.01 |
| En-Spm | 0 | 0 | 0.00 |
| MuDR-IS905 | 0 | 0 | 0.00 |
| PiggyBac | 0 | 0 | 0.00 |
| Tourist/Harbinger | 13 | 931 | 0.00 |
| [Other](http://www.ncbi.nlm.nih.gov/Taxonomy/Browser/wwwtax.cgi?lvl=0&id=32644) (Mirage P-element Transib) | 2 | 109 | 0.00 |
| Rolling-circles | 0 | 0 | 0.00 |
| [Unclassified](http://www.ncbi.nlm.nih.gov/Taxonomy/Browser/wwwtax.cgi?lvl=0&id=12908): | 6 | 536 | 0.00 |
| Total interspersed repeats: |  | 124336 | 0.18 |
| Small RNA: | 4644 | 590528 | 0.02 |
| [Satellites](http://www.ncbi.nlm.nih.gov/Taxonomy/Browser/wwwtax.cgi?lvl=0&id=12877): | 77 | 11338 | 0.02 |
| Simple repeats: | 13319 | 570227 | 0.89 |
| Low complexity: | 2759 | 140347 | 0.25 |

**Table S3.** Species and genes (accession numbers) used in the phylogenetic analysis. Modified from [[29](#_ENREF_29)].

| **Specie** | **LSU** | **ITS** | **TEF** |
| --- | --- | --- | --- |
| *Sedosporium apiospermum* HDO1 | --------- | -------- | ------ |
| *Sedosporium apiospermum* [IHEM14462](http://doi.org/10.1601/strainfinder?urlappend=%3Fid%3DIHEM+14462) | -------- | -------- | XM_016787526 |
| [*Wardomyces moseri*](http://www.ncbi.nlm.nih.gov/Taxonomy/Browser/wwwtax.cgi?lvl=0&id=1658444) CBS164.80 | [LN851049](https://www.ncbi.nlm.nih.gov/nuccore/LN851049) | [LN850995](https://www.ncbi.nlm.nih.gov/nuccore/LN850995) | [LN851100](https://www.ncbi.nlm.nih.gov/nuccore/LN851100) |
| [*Microascus albonigrescens*](http://www.ncbi.nlm.nih.gov/Taxonomy/Browser/wwwtax.cgi?lvl=0&id=186337) IHEM18560 | [LN851004](https://www.ncbi.nlm.nih.gov/nuccore/LN851004) | [LM652389](https://www.ncbi.nlm.nih.gov/nuccore/LM652389) | [LN851058](https://www.ncbi.nlm.nih.gov/nuccore/LN851058) |
| [*Trichurus spiralis*](http://www.ncbi.nlm.nih.gov/Taxonomy/Browser/wwwtax.cgi?lvl=0&id=186364) CBS635.78 | [LN851024](https://www.ncbi.nlm.nih.gov/nuccore/LN851024) | [LN850977](https://www.ncbi.nlm.nih.gov/nuccore/LN850977) | [LN851077](https://www.ncbi.nlm.nih.gov/nuccore/LN851077) |
| [*Microascus caviariformis*](http://www.ncbi.nlm.nih.gov/Taxonomy/Browser/wwwtax.cgi?lvl=0&id=186338) CBS536.87 | [LN851005](https://www.ncbi.nlm.nih.gov/nuccore/LN851005) | [LM652392](https://www.ncbi.nlm.nih.gov/nuccore/LM652392) | [LN851059](https://www.ncbi.nlm.nih.gov/nuccore/LN851059) |
| [*Scopulariopsis acremonium*](http://www.ncbi.nlm.nih.gov/Taxonomy/Browser/wwwtax.cgi?lvl=0&id=186355) MUCL9028 | [LN851001](https://www.ncbi.nlm.nih.gov/nuccore/LN851001) | [LM652456](https://www.ncbi.nlm.nih.gov/nuccore/LM652456) | [HG380362](https://www.ncbi.nlm.nih.gov/nuccore/HG380362) |
| [*Scopulariopsis acremonium*](http://www.ncbi.nlm.nih.gov/Taxonomy/Browser/wwwtax.cgi?lvl=0&id=186355) MUCL8274 | [LN851002](https://www.ncbi.nlm.nih.gov/nuccore/LN851002) | [LM652457](https://www.ncbi.nlm.nih.gov/nuccore/LM652457) | [LN851056](https://www.ncbi.nlm.nih.gov/nuccore/LN851056) |
| [*Scopulariopsis acremonium*](http://www.ncbi.nlm.nih.gov/Taxonomy/Browser/wwwtax.cgi?lvl=0&id=186355) MUCL8409 | [LN851003](https://www.ncbi.nlm.nih.gov/nuccore/LN851003) | [LM652458](https://www.ncbi.nlm.nih.gov/nuccore/LM652458) | [LN851057](https://www.ncbi.nlm.nih.gov/nuccore/LN851057) |
| [*Wardomyces*](http://www.ncbi.nlm.nih.gov/Taxonomy/Browser/wwwtax.cgi?lvl=0&id=186365) *litoralis* CBS119740 | [LN851055](https://www.ncbi.nlm.nih.gov/nuccore/LN851055) | [LN851000](https://www.ncbi.nlm.nih.gov/nuccore/LN851000) | [LN851107](https://www.ncbi.nlm.nih.gov/nuccore/LN851107) |
| [*Wardomyces*](http://www.ncbi.nlm.nih.gov/Taxonomy/Browser/wwwtax.cgi?lvl=0&id=186365) *inopinata* FMR10305 | [LN851054](https://www.ncbi.nlm.nih.gov/nuccore/LN851054) | [LM652498](https://www.ncbi.nlm.nih.gov/nuccore/LM652498) | [LN851106](https://www.ncbi.nlm.nih.gov/nuccore/LN851106) |
| [*Wardomyces*](http://www.ncbi.nlm.nih.gov/Taxonomy/Browser/wwwtax.cgi?lvl=0&id=186365) *inopinata* FMR10306 | [LN850956](https://www.ncbi.nlm.nih.gov/nuccore/LN850956) | [LN850955](https://www.ncbi.nlm.nih.gov/nuccore/LN850955) | [LN850957](https://www.ncbi.nlm.nih.gov/nuccore/LN850957) |
| [*Wardomyces humicola*](http://www.ncbi.nlm.nih.gov/Taxonomy/Browser/wwwtax.cgi?lvl=0&id=186367) CBS487.66 | [LM652554](https://www.ncbi.nlm.nih.gov/nuccore/LM652554) | [LM652497](https://www.ncbi.nlm.nih.gov/nuccore/LM652497) | [LN851103](https://www.ncbi.nlm.nih.gov/nuccore/LN851103) |
| [*Scopulariopsis humicola*](http://www.ncbi.nlm.nih.gov/Taxonomy/Browser/wwwtax.cgi?lvl=0&id=929264) FMR3993 | [LN851052](https://www.ncbi.nlm.nih.gov/nuccore/LN851052) | [LN850998](https://www.ncbi.nlm.nih.gov/nuccore/LN850998) | [LN851104](https://www.ncbi.nlm.nih.gov/nuccore/LN851104) |
| [*Wardomyces sp*.](http://www.ncbi.nlm.nih.gov/Taxonomy/Browser/wwwtax.cgi?lvl=0&id=186365) FMR13592 | [LN851053](https://www.ncbi.nlm.nih.gov/nuccore/LN851053) | [LN850999](https://www.ncbi.nlm.nih.gov/nuccore/LN850999) | [LN851105](https://www.ncbi.nlm.nih.gov/nuccore/LN851105) |
| [*Microascus giganteus*](http://www.ncbi.nlm.nih.gov/Taxonomy/Browser/wwwtax.cgi?lvl=0&id=129315) CBS746.69 | [LN851045](https://www.ncbi.nlm.nih.gov/nuccore/LN851045) | [LM652411](https://www.ncbi.nlm.nih.gov/nuccore/LM652411) | [LN851096](https://www.ncbi.nlm.nih.gov/nuccore/LN851096) |
| [*Wardomyces humicola*](http://www.ncbi.nlm.nih.gov/Taxonomy/Browser/wwwtax.cgi?lvl=0&id=186367) CBS369.62 | [LN851046](https://www.ncbi.nlm.nih.gov/nuccore/LN851046) | [LN850993](https://www.ncbi.nlm.nih.gov/nuccore/LN850993) | [LN851097](https://www.ncbi.nlm.nih.gov/nuccore/LN851097) |
| [*Wardomyces papillatus*](http://www.ncbi.nlm.nih.gov/Taxonomy/Browser/wwwtax.cgi?lvl=0&id=1658446) CBS112.65 | [LN851051](https://www.ncbi.nlm.nih.gov/nuccore/LN851051) | [LN850997](https://www.ncbi.nlm.nih.gov/nuccore/LN850997) | [LN851102](https://www.ncbi.nlm.nih.gov/nuccore/LN851102) |
| [*Wardomyces columbinus*](http://www.ncbi.nlm.nih.gov/Taxonomy/Browser/wwwtax.cgi?lvl=0&id=1658442) CBS233.66 | [LN851039](https://www.ncbi.nlm.nih.gov/nuccore/LN851039) | [LN850990](https://www.ncbi.nlm.nih.gov/nuccore/LN850990) | [LN851092](https://www.ncbi.nlm.nih.gov/nuccore/LN851092) |
| [*Wardomyces dimerus*](http://www.ncbi.nlm.nih.gov/Taxonomy/Browser/wwwtax.cgi?lvl=0&id=1658443) CBS235.66 | [LN851040](https://www.ncbi.nlm.nih.gov/nuccore/LN851040) | [LN850991](https://www.ncbi.nlm.nih.gov/nuccore/LN850991) | [LN851093](https://www.ncbi.nlm.nih.gov/nuccore/LN851093) |
| [*Wardomyces simplex*](http://www.ncbi.nlm.nih.gov/Taxonomy/Browser/wwwtax.cgi?lvl=0&id=186370) CBS546.69 | [LN851041](https://www.ncbi.nlm.nih.gov/nuccore/LN851041) | [LM652379](https://www.ncbi.nlm.nih.gov/nuccore/LM652379) | [LN851094](https://www.ncbi.nlm.nih.gov/nuccore/LN851094) |
| [*Wardomyces columbinus*](http://www.ncbi.nlm.nih.gov/Taxonomy/Browser/wwwtax.cgi?lvl=0&id=1658442) CBS230.82 | [LN851038](https://www.ncbi.nlm.nih.gov/nuccore/LN851038) | [LN850989](https://www.ncbi.nlm.nih.gov/nuccore/LN850989) | [LN851091](https://www.ncbi.nlm.nih.gov/nuccore/LN851091) |
| [*Wardomyces aggregatus*](http://www.ncbi.nlm.nih.gov/Taxonomy/Browser/wwwtax.cgi?lvl=0&id=1526918) CBS251.69 | [LN851037](https://www.ncbi.nlm.nih.gov/nuccore/LN851037) | [LM652378](https://www.ncbi.nlm.nih.gov/nuccore/LM652378) | [LN851090](https://www.ncbi.nlm.nih.gov/nuccore/LN851090) |
| [*Wardomyces ovalis*](http://www.ncbi.nlm.nih.gov/Taxonomy/Browser/wwwtax.cgi?lvl=0&id=1658445) CBS234.66 | [LN851050](https://www.ncbi.nlm.nih.gov/nuccore/LN851050) | [LN850996](https://www.ncbi.nlm.nih.gov/nuccore/LN850996) | [LN851101](https://www.ncbi.nlm.nih.gov/nuccore/LN851101) |
| [*Wardomyces inflatus*](http://www.ncbi.nlm.nih.gov/Taxonomy/Browser/wwwtax.cgi?lvl=0&id=186368) CBS367.62 | [LN851048](https://www.ncbi.nlm.nih.gov/nuccore/LN851048) | [LN850994](https://www.ncbi.nlm.nih.gov/nuccore/LN850994) | [LN851099](https://www.ncbi.nlm.nih.gov/nuccore/LN851099) |
| [*Wardomyces anomalus*](http://www.ncbi.nlm.nih.gov/Taxonomy/Browser/wwwtax.cgi?lvl=0&id=186366) CBS299.61 | [LN851044](https://www.ncbi.nlm.nih.gov/nuccore/LN851044) | [LN850992](https://www.ncbi.nlm.nih.gov/nuccore/LN850992) | [LN851095](https://www.ncbi.nlm.nih.gov/nuccore/LN851095) |
| [*Wardomyces hughesii*](http://www.ncbi.nlm.nih.gov/Taxonomy/Browser/wwwtax.cgi?lvl=0&id=1658450) CBS216.61 | [LN851047](https://www.ncbi.nlm.nih.gov/nuccore/LN851047) | [LM652496](https://www.ncbi.nlm.nih.gov/nuccore/LM652496) | [LN851098](https://www.ncbi.nlm.nih.gov/nuccore/LN851098) |
| [*Trichurus dendrocephalus*](http://www.ncbi.nlm.nih.gov/Taxonomy/Browser/wwwtax.cgi?lvl=0&id=644594) CBS528.85 | [LN851013](https://www.ncbi.nlm.nih.gov/nuccore/LN851013) | [LN850966](https://www.ncbi.nlm.nih.gov/nuccore/LN850966) | [LN851067](https://www.ncbi.nlm.nih.gov/nuccore/LN851067) |
| [*Doratomyces asperulus*](http://www.ncbi.nlm.nih.gov/Taxonomy/Browser/wwwtax.cgi?lvl=0&id=1658437) CBS187.78 | [LN851033](https://www.ncbi.nlm.nih.gov/nuccore/LN851033) | [LN850986](https://www.ncbi.nlm.nih.gov/nuccore/LN850986) | [LN851086](https://www.ncbi.nlm.nih.gov/nuccore/LN851086) |
| [*Doratomyces purpureofuscus*](http://www.ncbi.nlm.nih.gov/Taxonomy/Browser/wwwtax.cgi?lvl=0&id=186353) UAMH9209 | [LN851018](https://www.ncbi.nlm.nih.gov/nuccore/LN851018) | [LN850971](https://www.ncbi.nlm.nih.gov/nuccore/LN850971) | [LN851072](https://www.ncbi.nlm.nih.gov/nuccore/LN851072) |
| [*Doratomyces purpureofuscus*](http://www.ncbi.nlm.nih.gov/Taxonomy/Browser/wwwtax.cgi?lvl=0&id=186353) CBS 157 57 | [LN851031](https://www.ncbi.nlm.nih.gov/nuccore/LN851031) | [LN850984](https://www.ncbi.nlm.nih.gov/nuccore/LN850984) | [LN851084](https://www.ncbi.nlm.nih.gov/nuccore/LN851084) |
| [*Trichurus spiralis*](http://www.ncbi.nlm.nih.gov/Taxonomy/Browser/wwwtax.cgi?lvl=0&id=186364) CBS 131 08 | [LN851021](https://www.ncbi.nlm.nih.gov/nuccore/LN851021) | [LN850974](https://www.ncbi.nlm.nih.gov/nuccore/LN850974) | -------- |
| [*Doratomyces microsporus*](http://www.ncbi.nlm.nih.gov/Taxonomy/Browser/wwwtax.cgi?lvl=0&id=186352) UTHSCDI1464 | [LN851027](https://www.ncbi.nlm.nih.gov/nuccore/LN851027) | [LN850980](https://www.ncbi.nlm.nih.gov/nuccore/LN850980) | [LN851080](https://www.ncbi.nlm.nih.gov/nuccore/LN851080) |
| [*Doratomyces* sp.](http://www.ncbi.nlm.nih.gov/Taxonomy/Browser/wwwtax.cgi?lvl=0&id=1856739) UTHSCDI1469 | [LN851028](https://www.ncbi.nlm.nih.gov/nuccore/LN851028) | [LN850981](https://www.ncbi.nlm.nih.gov/nuccore/LN850981) | [LN851081](https://www.ncbi.nlm.nih.gov/nuccore/LN851081) |
| [*Trichurus terrophilus*](http://www.ncbi.nlm.nih.gov/Taxonomy/Browser/wwwtax.cgi?lvl=0&id=644595) CBS368.53 | [LN851023](https://www.ncbi.nlm.nih.gov/nuccore/LN851023) | [LN850976](https://www.ncbi.nlm.nih.gov/nuccore/LN850976) | [LN851076](https://www.ncbi.nlm.nih.gov/nuccore/LN851076) |
| [*Doratomyces purpureofuscus*](http://www.ncbi.nlm.nih.gov/Taxonomy/Browser/wwwtax.cgi?lvl=0&id=186353) UTHSCDI1471 | [LN851029](https://www.ncbi.nlm.nih.gov/nuccore/LN851029) | [LN850982](https://www.ncbi.nlm.nih.gov/nuccore/LN850982) | [LN851082](https://www.ncbi.nlm.nih.gov/nuccore/LN851082) |
| [*Doratomyces sp*.](http://www.ncbi.nlm.nih.gov/Taxonomy/Browser/wwwtax.cgi?lvl=0&id=1856739) UTHSC1463 | [LN851026](https://www.ncbi.nlm.nih.gov/nuccore/LN851026) | [LN850979](https://www.ncbi.nlm.nih.gov/nuccore/LN850979) | [LN851079](https://www.ncbi.nlm.nih.gov/nuccore/LN851079) |
| [*Trichurus spiralis*](http://www.ncbi.nlm.nih.gov/Taxonomy/Browser/wwwtax.cgi?lvl=0&id=186364) UAMH3585 | [LN851025](https://www.ncbi.nlm.nih.gov/nuccore/LN851025) | [LN850978](https://www.ncbi.nlm.nih.gov/nuccore/LN850978) | [LN851078](https://www.ncbi.nlm.nih.gov/nuccore/LN851078) |
| [*Trichurus cylindricus*](http://www.ncbi.nlm.nih.gov/Taxonomy/Browser/wwwtax.cgi?lvl=0&id=1658440) UAMH1348 | [LN851012](https://www.ncbi.nlm.nih.gov/nuccore/LN851012) | [LN850965](https://www.ncbi.nlm.nih.gov/nuccore/LN850965) | [LN851066](https://www.ncbi.nlm.nih.gov/nuccore/LN851066) |
| [*Doratomyces asperulus*](http://www.ncbi.nlm.nih.gov/Taxonomy/Browser/wwwtax.cgi?lvl=0&id=1658437) CBS127.22 | [LN851006](https://www.ncbi.nlm.nih.gov/nuccore/LN851006) | [LN850959](https://www.ncbi.nlm.nih.gov/nuccore/LN850959) | [LN851060](https://www.ncbi.nlm.nih.gov/nuccore/LN851060) |
| [*Doratomyces asperulus*](http://www.ncbi.nlm.nih.gov/Taxonomy/Browser/wwwtax.cgi?lvl=0&id=1658437) CBS582.71 | [LN851007](https://www.ncbi.nlm.nih.gov/nuccore/LN851007) | [LN850960](https://www.ncbi.nlm.nih.gov/nuccore/LN850960) | [LN851061](https://www.ncbi.nlm.nih.gov/nuccore/LN851061) |
| [*Doratomyces sp*.](http://www.ncbi.nlm.nih.gov/Taxonomy/Browser/wwwtax.cgi?lvl=0&id=1856739) UTHSCDI1462 | [LN851008](https://www.ncbi.nlm.nih.gov/nuccore/LN851008) | [LN850961](https://www.ncbi.nlm.nih.gov/nuccore/LN850961) | [LN851062](https://www.ncbi.nlm.nih.gov/nuccore/LN851062) |
| [*Doratomyces sp*.](http://www.ncbi.nlm.nih.gov/Taxonomy/Browser/wwwtax.cgi?lvl=0&id=1856739) UTHSCDI1465 | [LN851009](https://www.ncbi.nlm.nih.gov/nuccore/LN851009) | [LN850962](https://www.ncbi.nlm.nih.gov/nuccore/LN850962) | [LN851063](https://www.ncbi.nlm.nih.gov/nuccore/LN851063) |
| [*Doratomyces purpureofuscus*](http://www.ncbi.nlm.nih.gov/Taxonomy/Browser/wwwtax.cgi?lvl=0&id=186353) CBS523.63 | [LN851014](https://www.ncbi.nlm.nih.gov/nuccore/LN851014) | [LN850967](https://www.ncbi.nlm.nih.gov/nuccore/LN850967) | [LN851068](https://www.ncbi.nlm.nih.gov/nuccore/LN851068) |
| [*Doratomyces microsporus*](http://www.ncbi.nlm.nih.gov/Taxonomy/Browser/wwwtax.cgi?lvl=0&id=186352) UAMH9365 | [LN851015](https://www.ncbi.nlm.nih.gov/nuccore/LN851015) | [LN850968](https://www.ncbi.nlm.nih.gov/nuccore/LN850968) | [LN851069](https://www.ncbi.nlm.nih.gov/nuccore/LN851069) |
| [*Doratomyces nanus*](http://www.ncbi.nlm.nih.gov/Taxonomy/Browser/wwwtax.cgi?lvl=0&id=644570) UAMH9126 | [LN851017](https://www.ncbi.nlm.nih.gov/nuccore/LN851017) | [LN850970](https://www.ncbi.nlm.nih.gov/nuccore/LN850970) | [LN851071](https://www.ncbi.nlm.nih.gov/nuccore/LN851071) |
| [*Doratomyces stemonitis*](http://www.ncbi.nlm.nih.gov/Taxonomy/Browser/wwwtax.cgi?lvl=0&id=186354) CBS289.66 | [LN851032](https://www.ncbi.nlm.nih.gov/nuccore/LN851032) | [LN850985](https://www.ncbi.nlm.nih.gov/nuccore/LN850985) | [LN851085](https://www.ncbi.nlm.nih.gov/nuccore/LN851085) |
| [*Doratomyces stemonitis*](http://www.ncbi.nlm.nih.gov/Taxonomy/Browser/wwwtax.cgi?lvl=0&id=186354) | [LN850952](https://www.ncbi.nlm.nih.gov/nuccore/LN850952) | [LN850951](https://www.ncbi.nlm.nih.gov/nuccore/LN850951) | [LN850953](https://www.ncbi.nlm.nih.gov/nuccore/LN850953) |
| [*Doratomyces stemonitis*](http://www.ncbi.nlm.nih.gov/Taxonomy/Browser/wwwtax.cgi?lvl=0&id=186354) CBS180.35 | [LN851019](https://www.ncbi.nlm.nih.gov/nuccore/LN851019) | [LN850972](https://www.ncbi.nlm.nih.gov/nuccore/LN850972) | [LN851073](https://www.ncbi.nlm.nih.gov/nuccore/LN851073) |
| [*Doratomyces stemonitis*](http://www.ncbi.nlm.nih.gov/Taxonomy/Browser/wwwtax.cgi?lvl=0&id=186354) UAMH1532 | [LN851020](https://www.ncbi.nlm.nih.gov/nuccore/LN851020) | [LN850973](https://www.ncbi.nlm.nih.gov/nuccore/LN850973) | [LN851074](https://www.ncbi.nlm.nih.gov/nuccore/LN851074) |
| [*Doratomyces nanus*](http://www.ncbi.nlm.nih.gov/Taxonomy/Browser/wwwtax.cgi?lvl=0&id=644570) CBS191.61 | [LN851016](https://www.ncbi.nlm.nih.gov/nuccore/LN851016) | [LN850969](https://www.ncbi.nlm.nih.gov/nuccore/LN850969) | [LN851070](https://www.ncbi.nlm.nih.gov/nuccore/LN851070) |
| [*Doratomyces columnaris*](http://www.ncbi.nlm.nih.gov/Taxonomy/Browser/wwwtax.cgi?lvl=0&id=644569) CBS159.66 | [LN851010](https://www.ncbi.nlm.nih.gov/nuccore/LN851010) | [LN850963](https://www.ncbi.nlm.nih.gov/nuccore/LN850963) | [LN851064](https://www.ncbi.nlm.nih.gov/nuccore/LN851064) |
| [*Microascus singularis*](http://www.ncbi.nlm.nih.gov/Taxonomy/Browser/wwwtax.cgi?lvl=0&id=186346) CBS249.64 | [LN851034](https://www.ncbi.nlm.nih.gov/nuccore/LN851034) | [LN850987](https://www.ncbi.nlm.nih.gov/nuccore/LN850987) | [LN851087](https://www.ncbi.nlm.nih.gov/nuccore/LN851087) |
| [*Microascus singularis*](http://www.ncbi.nlm.nih.gov/Taxonomy/Browser/wwwtax.cgi?lvl=0&id=186346) CBS505.66 | [LN851036](https://www.ncbi.nlm.nih.gov/nuccore/LN851036) | [LN850988](https://www.ncbi.nlm.nih.gov/nuccore/LN850988) | [LN851089](https://www.ncbi.nlm.nih.gov/nuccore/LN851089) |
| [*Microascus singularis*](http://www.ncbi.nlm.nih.gov/Taxonomy/Browser/wwwtax.cgi?lvl=0&id=186346) CBS414.64 | [LN851035](https://www.ncbi.nlm.nih.gov/nuccore/LN851035) | [LM652442](https://www.ncbi.nlm.nih.gov/nuccore/LM652442) | [LN851088](https://www.ncbi.nlm.nih.gov/nuccore/LN851088) |
| [*Scopulariopsis brevicaulis*](http://www.ncbi.nlm.nih.gov/Taxonomy/Browser/wwwtax.cgi?lvl=0&id=561152) MUCL40726 | [LN851042](https://www.ncbi.nlm.nih.gov/nuccore/LN851042) | [LM652465](https://www.ncbi.nlm.nih.gov/nuccore/LM652465) | [HG380363](https://www.ncbi.nlm.nih.gov/nuccore/HG380363) |
| [*Microascus longirostris*](http://www.ncbi.nlm.nih.gov/Taxonomy/Browser/wwwtax.cgi?lvl=0&id=186343) CBS196.61 | [LN851043](https://www.ncbi.nlm.nih.gov/nuccore/LN851043) | [LM652421](https://www.ncbi.nlm.nih.gov/nuccore/LM652421) | [LM652566](https://www.ncbi.nlm.nih.gov/nuccore/LM652566) |
